# Polymorphisms in the Mannose-Binding Lectin Gene are Associated with Defective Mannose-Binding Lectin Functional Activity in Crohn’s Disease Patients

**DOI:** 10.1038/srep29636

**Published:** 2016-07-12

**Authors:** Laura Choteau, Francis Vasseur, Frederic Lepretre, Martin Figeac, Corine Gower-Rousseau, Laurent Dubuquoy, Daniel Poulain, Jean-Frederic Colombel, Boualem Sendid, Samir Jawhara

**Affiliations:** 1 INSERM, U995, F-59000 Lille, France; 2 University Lille2, U995-LIRIC, Lille Inflammation Research International Centre, F-59000 Lille, France; 3 CHU Lille, Service de Parasitologie Mycologie, Pôle de Biologie Pathologie Génétique, F-59000 Lille, France; 4 Université Lille Nord de France, Unité de Biostatistique, EA 2694, F-59000 Lille, France; 5 Genetic platform, F-59000 Lille, France; 6 Department of Gastroenterology, Icahn School of Medicine at Mount Sinai, New York, NY, USA

## Abstract

Mannose-binding lectin, together with mannose-associated serine proteases, activates the lectin pathway of the complement system and subsequent inflammatory mechanisms. An association between mannose-binding lectin deficiency and anti-*Saccharomyces cerevisiae* antibody levels is observed in Crohn’s disease and this deficiency is frequently associated with a severe Crohn’s disease phenotype. In the present study, we assessed the relationship between serum concentrations of mannose-binding lectin, mannose-binding lectin functional activity, *MBL2* and *NOD2* polymorphisms, anti-*S. cerevisiae* antibody levels and clinical Crohn’s disease phenotype in 69 Crohn’s disease patients and 30 age- and sex-matched healthy controls. The results show that the *MBL2* variant rs5030737 at codon 52 was associated with a low level of mannose-binding lectin and impaired mannose-binding lectin–mannose-associated serine protease (MBL-MASP) functional activity in Crohn’s disease patients. This *MBL2* variant was also associated with a higher level of anti-*S. cerevisiae* antibodies. In addition, the *NOD2* variant rs2066844, which is associated with susceptibility to Crohn’s disease, was significantly correlated with an impairment in MBL-MASP functional activity. These results provide evidence that Crohn’s disease patients have an impairment in MBL-MASP functional activity and that this defect is associated with *MBL2* and *NOD2* variants.

Inflammatory bowel disease is a chronic inflammatory disease of the gastrointestinal tract which includes Crohn’s disease and ulcerative colitis^1^. Although the aetiology of irritable bowel disease is unclear several studies have showed that genetic susceptibility, the microbiota, environment and immune system are all involved in its pathogenesis^2^,^3^,^4^. Studies in twins have provided the best evidence for genetic predisposition to irritable bowel disease^5^. Relatives of patients with Crohn’s disease have a higher risk of developing irritable bowel disease than those of patients with ulcerative colitis^5^. In addition to the clinical characteristics of irritable bowel disease such as patient age at diagnosis, disease location and disease behaviour, serological markers, in particular anti-*Saccharomyces cerevisiae* antibodies, can improve the accuracy of diagnosis of irritable bowel disease. Anti-*S. cerevisiae* antibodies have the highest sensitivity as serological markers of Crohn’s disease^6^. Plevy *et al*. showed that incorporating a combination of serological, genetic and inflammatory markers into a diagnostic algorithm improved the accuracy of diagnosis of irritable bowel disease^7^. The genetic association of NOD2/CARD15 with Crohn’s disease has established a critical link between innate immune cells, the intestinal epithelium and development of the disease^8^. In addition, mannose-binding lectin is another critical protein, which has a major role in the innate immune defence against pathogens and promotes intestinal homeostasis^9^,^10^. This lectin circulates in the blood as a complex with mannose-associated serine proteases. The mannose-binding lectin–mannose-associated serine protease (MBL-MASP) complex activates the lectin complement pathway after recognition of microorganisms through the carbohydrate recognition domain^11^,^12^. The carbohydrate recognition domain of mannose-binding lectin senses polysaccharide patterns such as D-mannose, L-fucose and N-acetylglucosamine on several clinically relevant pathogens^13^. This results in activation of MASP-1 and MASP-2 leading to the complement cascade and to pathogen neutralisation. It has been shown that MASP-1 is the main activator of MASP-2^14^,^15^. Both MASP-1 and MASP-2 can form co-complexes with mannose-binding lectin and are crucial to lectin pathway activation. They are also able to cleave prothrombin, promoting clot formation^16^,^17^,^18^.

It has been reported that single nucleotide polymorphisms located within the promoter region and exon 1 of the *MBL2* gene are correlated with mannose-binding lectin serum levels and, consequently, are associated with a higher risk of developing infectious disease. Several studies have shown an association between mutations in the *MBL2* gene and Crohn’s disease^9^,^19^. Seibold *et al*. suggested that an impaired innate immune system defined by mannose-binding lectin deficiency may lead to increased reactivity to mannan antigens in the microbial cell wall and that this enhanced mannan exposure contributes to the generation of anti-*S. cerevisiae* antibodies in patients with Crohn’s disease^20^,^21^. Uemura *et al*. showed that mannose-binding lectin is expressed in epithelial cells in the murine small intestine^22^. Recently, we showed that mannose-binding lectin is produced locally by human colon epithelial cells in response to intestinal inflammation where it plays a crucial role in the mucosal antifungal defence and intestinal homeostasis^10^. Although the association between mannose-binding lectin serum concentrations and *MBL2* gene mutations in Crohn’s disease patients has been studied previously, the functional activity of the MBL-MASP complex has not yet been investigated in any clinical cohort of Crohn’s disease patients.

The present study aimed to investigate the relationship between mannose-binding lectin serum concentrations, mannose-binding lectin functional activity, *MBL2*, *MASP1*, *MASP2* and *NOD2* variants, anti-*S. cerevisiae* antibody levels and clinical Crohn’s disease phenotype in a cohort of Crohn’s disease patients in comparison with healthy subjects.

## Results

### Association between mannose-binding lectin serum concentrations and clinical phenotype of Crohn’s disease

**Figure 1:**
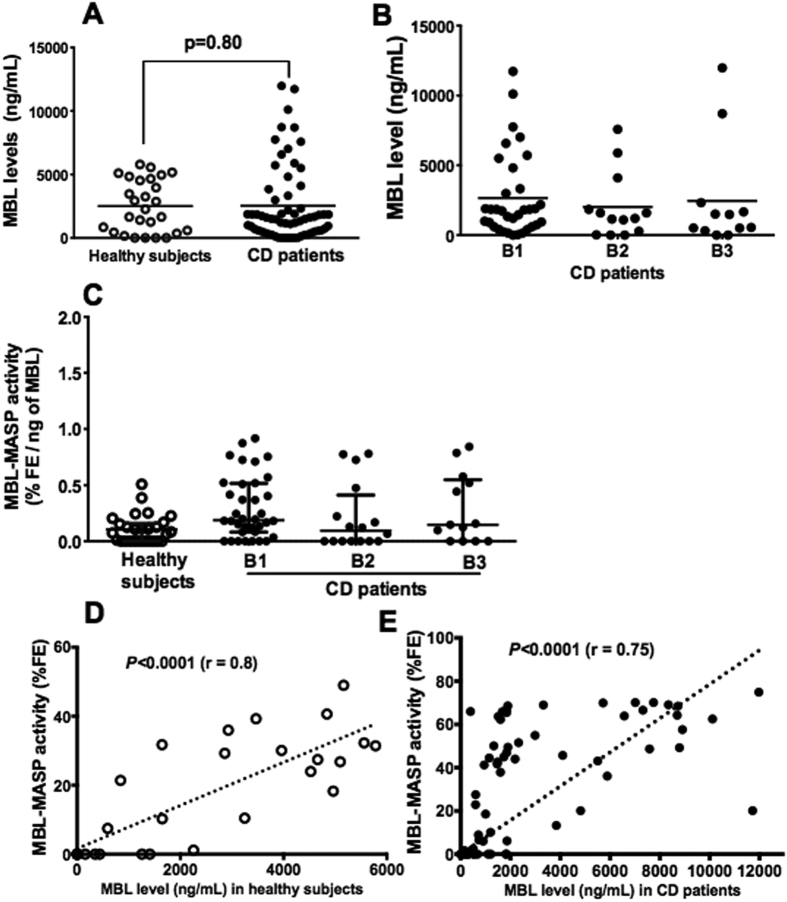
Relationship between functional activity of the MBL-MASP complex and mannose-binding lectin concentration. **(A)** Mannose-binding lectin level was determined in 30 healthy control subjects and 69 Crohn’s disease patients. There was no significant difference between the two groups. **(B)** No significant difference was found between the concentration of mannose-binding lectin and clinical phenotype of Crohn’s disease. Scatter plots of these data with the median line are shown. Mannose-binding lectin concentration was determined in duplicate for each sample. **(C)** Functional activity of the MBL-MASP complex was determined in 30 healthy controls (open dot) and 69 Crohn’s disease patients (black dot). Data are the mean ± SD of two independent experiments. **(D,E)** Correlation between functional activity of the MBL-MASP complex and mannose-binding lectin concentration in 30 healthy controls (*P* < 0.0001, R = 0.8) and 69 Crohn’s disease patients (*P* < 0.0001, R = 0.75). AU, Arbitrary units.

Serum concentrations of mannose-binding lectin were not statistically different between Crohn’s disease patients and healthy controls although a slightly elevated mannose-binding lectin level was observed in Crohn’s disease patients (*P* = 0.8). The concentration of mannose-binding lectin was not associated to the clinical phenotype of the disease (B1, B2, or B3) (Fig. 1A,B).

### Association between functional activity of the MBL-MASP complex and clinical phenotype of Crohn’s disease

To assess whether mannose-binding lectin is able to bind to mannose-associated serine proteases and then to activate the complement system in the serum of Crohn’s disease patients we explored the functional activity of the MBL-MASP complex. The functional activity assay was based on the ability of mannose-binding lectin to bind to *S. cerevisiae* mannan and the ability of mannose-associated serine proteases to cleave the fluoregenic substrate of thrombin.

The functional activity of the MBL-MASP complex was measured in 69 Crohn’s disease and 30 healthy control sera. No functional activity of the MBL-MASP complex was detected in either Crohn’s disease patients or healthy control subjects when the mannose-binding lectin level was <500 ng/mL (Fig. 1C). Furthermore, no significant difference in functional activity of the MBL-MASP complex was observed between healthy controls and Crohn’s disease patients. Increased functional activity of the MBL-MASP complex was correlated with the mannose-binding lectin serum level in both healthy controls (*P* < 0.0001, r = 0.8, Fig. 1D) and Crohn’s disease patients (*P* < 0.0001, r = 0.75, Fig. 1E), particularly when the mannose-binding lectin serum concentrations were >500 ng/mL. This suggests that the serum concentration of mannose-binding lectin has an important impact on mannose-binding lectin functional activity.

### Association between high anti-*S. cerevisiae* antibody levels and the B2 phenotype

**Figure 2:**
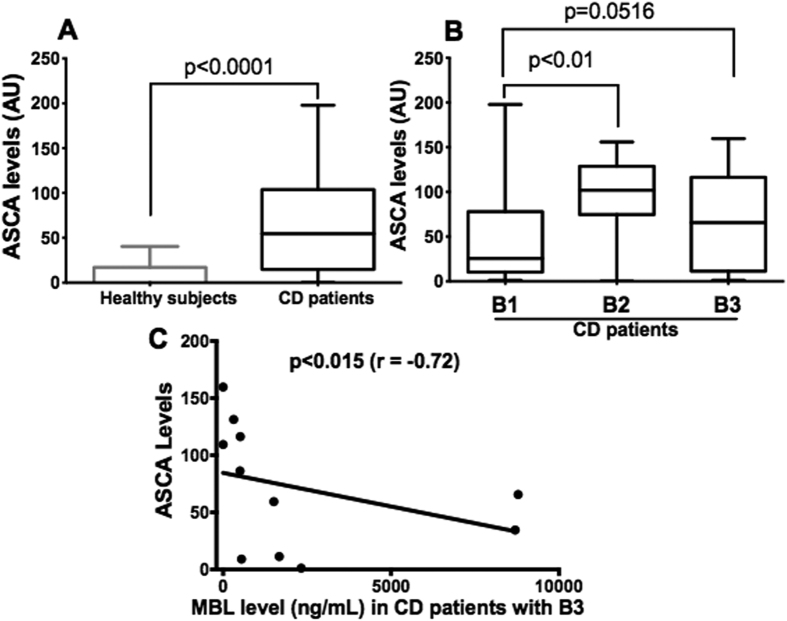
Anti-*S. cerevisiae* antibody levels in healthy control subjects and Crohn’s disease patients. **(A)** Anti-*S. cerevisiae* antibody level was increased in Crohn’s disease patients when compared to healthy controls (*P* < 0.0001). Scatter plots of these data with the median line are shown. **(B)** Crohn’s disease patients with the clinical phenotype B2 had higher anti-*S. cerevisiae* antibody levels than those with B1 (*P* < 0.01) and there was a tendency for Crohn’s disease patients with B3 to have higher levels than patients with B1 (*P* = 0.0516). **(C)** Correlation between anti-*S. cerevisiae* antibody levels and mannose-binding lectin concentrations in Crohn’s disease patients with B3 (*P* < 0.015, r = −0.72). The results are expressed in arbitrary units (AU).

Anti-*S. cerevisiae* antibody levels were significantly higher in Crohn’s disease patients compared to healthy controls (*P* < 0.0001) (Fig. 2A). Furthermore, anti-*S. cerevisiae* antibody levels were significantly elevated in Crohn’s disease patients with the B2 phenotype compared to patients with the B1 phenotype (*P* < 0.01) and there was also a tendency towards elevated anti-*S. cerevisiae* antibody levels in Crohn’s disease patients with the B3 phenotype (*P* = 0.0516) (Fig. 2B). Mannose-binding lectin levels were inversely correlated with anti-*S. cerevisiae* antibody levels in Crohn’s disease patients with severe clinical phenotypes (*P* < 0.015, r = −0.72) (Fig. 2C).

### Relationship between the *MBL2* mutation at codon 52, mannose-binding lectin serum concentrations, MBL-MASP functional activity and anti-*S. cerevisiae* antibody levels in Crohn’s disease patients

**Figure 3:**
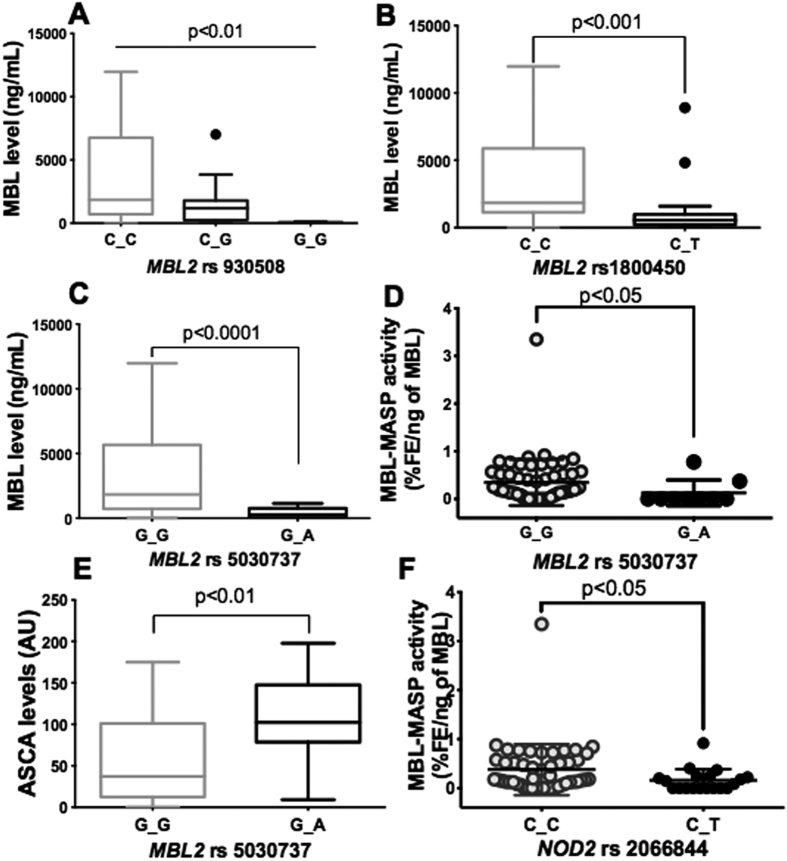
Association between *MBL2* and *NOD2* polymorphisms, mannose-binding lectin concentration, functional activity of the MBL-MASP complex and anti-*S. cerevisiae* antibody levels in Crohn’s disease patients (A–C). Mannose-binding lectin concentration was significantly associated with rs930508 (wild-type C/C heterozygous C/G or homozygous G/G; *P* < 0.01), rs1800450 (wild-type C/C, heterozygote C/T; *P* < 0.001) and rs5030737 (wild-type G/G, heterozygote G/A; *P* < 0.0001) of *MBL2* polymorphisms in Crohn’s disease patients. (**D**) Functional activity of the MBL-MASP complex was significantly associated with the rs5030737 *MBL2* polymorphism in Crohn’s disease patients (wild-type G/G, heterozygote G/A; *P* < 0.05). (**E**) Relationship between the rs5030737 *MBL2* polymorphism and anti-*S. cerevisiae* antibody levels in Crohn’s disease patients. Anti-*S. cerevisiae* antibody level was significantly higher in heterozygous Crohn’s disease patients (G/A; *n* = 9) than in wild-type patients (G/G; *n* = 60) for the rs5030737 *MBL2* variant (*P* < 0.01). (**F**) Association between functional activity of the MBL-MASP complex and the *NOD2* polymorphism in Crohn’s disease patients. Functional activity of the MBL-MASP1 complex was significantly higher in heterozygous Crohn’s disease patients (C/T; *n* = 20) when compared to wild-type patients (C/C; *n* = 50) for the rs2066844 *NOD2* variant (*P* < 0.05).

To explore whether *MBL2* polymorphisms are associated with susceptibility to Crohn’s disease and to its clinical and serological manifestations, the *MBL2* gene and its promoter were genotyped in 69 Crohn’s disease patients and 30 healthy controls. Two polymorphisms of the *MBL2* gene were identified: rs930508 and rs1800450, which were associated with significant mannose-binding lectin deficiency (*P* < 0.01 and *P* < 0.0001, respectively; Fig. 3A,B). The polymorphism rs5030737 (codon 52) was associated with a decrease in mannose-binding lectin serum levels (*P* < 0.0001) and a low level of MBL-MASP functional activity (*P* < 0.05) in Crohn’s disease patients (Fig. 3C,D). In addition, the polymorphism rs5030737 was associated with significantly increased levels of anti-*S. cerevisiae* antibodies (*P* < 0.01) in Crohn’s disease patients (Fig. 3E). In terms of the association between mannose-binding lectin polymorphisms and clinical phenotype of Crohn’s disease, the polymorphism rs5030737 was more common in Crohn’s disease patients with B2 and B3 phenotypes than in those with B1 (Table 1). 13% of Crohn’s disease patients had heterozygous mutations for the rs5030737 variant, which represent 77.7% for B2 and B3 clinical phenotypes vs. 22.3% for B1.

### Association between the *NOD2* polymorphism and functional activity of the MBL-MASP complex

To investigate an additional genetic marker for Crohn’s disease, the *NOD2* gene was genotyped in 69 Crohn’s disease patients and 30 healthy controls. A significant association was found between the rs2066847 polymorphism and Crohn’s disease (*P* = 0.0177) and there was a tendency for the rs2066844 polymorphism to be associated with the disease (*P* = 0.0518). In terms of the association between clinical phenotype and the *NOD2* polymorphism, the rs2066847 variant was found in 15 Crohn’s disease patients with clinical phenotypes B1 (33.3%), B2 (40.1%) and B3 (26.6%), respectively, carrying the heterozygous mutation C_C (Table 2) and in two Crohn’s disease patients carrying the homozygous mutation (both B3). The *NOD2* variant rs2066844 was found in 19 Crohn’s disease patients (heterozygous mutations C_T) with B1 (73.3%), B2 (15.8%) and B3 (10.5%), respectively (Table 2).

While the *NOD2* variant rs2066844 was not associated with mannose-binding lectin serum levels, the polymorphism was associated with functional activity of the MBL-MASP complex (Fig. 3F). Significantly lower functional activity of the MBL-MASP complex was observed in Crohn’s disease patients carrying the *NOD2* variant rs2066844 (R702W) compared to those with the *NOD2* wild-type (*P* < 0.05) (Fig. 3F). In addition to genotyping the *MBL2* and *NOD2* genes, the MASP1 gene was also genotyped in this study. No association was found between *MASP1* and Crohn’s disease (Table 3).

## Discussion

The MBL-MASP complex is an activator of the lectin pathway of the complement system and subsequent inflammatory mechanisms^23^. In the present study, mannose-binding lectin serum levels did not vary significantly between Crohn’s disease patients and healthy controls. In addition, mannose-binding lectin serum levels were not associated with the clinical phenotype of Crohn’s disease. These data are consistent with previous clinical studies that show the absence of mannose-binding lectin level changes in Crohn’s disease patients^24^,^25^. Experimental studies showed that MASP-1 and MASP-2 are involved in blood coagulation^12^,^26^,^27^. Hajela *et al*. showed that soluble native human MASP-1 has a thrombin-like substrate specificity, cleaving and activating the coagulation proteins Factor XIII and fibrinogen^12^,^18^. In addition, MASP-2 is capable of generating thrombin via prothrombin^17^.

In the present study, we explored the functional activity of the MBL-MASP complex using a coagulation protease substrate. The test was based on the ability of mannose-binding lectin to bind to *S. cerevisiae* mannan through its carbohydrate recognition domain and the ability of the mannose-associated serine protease-associated collagen-like domain of mannose-binding lectin to cleave the fluorogenic protease substrate^27^. We observed a significant correlation between mannose-binding lectin serum concentrations and functional activity of the MBL-MASP complex in both Crohn’s disease patients and healthy controls. However, a mannose-binding lectin concentration of <500 ng/mL was associated with an impairment in MBL-MASP functional activity and the absence of enzymatic activity in serum samples from Crohn’s disease patients. To corroborate the functional activity observed in the fluorogenic thrombin assay, two other assays were performed. The first was based on cleavage of complement C4 protein to C4b fragments and the second on the activation of platelets that had been exposed to the MBL-MASP complex trapped on mannan-coated plates. In these assays, we found a significant correlation between mannose-binding lectin concentrations and functional activity of the MBL-MASP complex in both Crohn’s disease patients and healthy controls. These results are consistent with those from the fluorogenic thrombin assay (Supplementary data).

Anti-*S. cerevisiae* antibodies are important serological markers that can help to differentiate Crohn’s disease from ulcerative colitis^6^. In the present study, anti-*S. cerevisiae* antibody levels were significantly elevated in Crohn’s disease patients with stricture formation and penetrating disease complications indicating that higher anti-*S. cerevisiae* antibody levels could be a predictive marker of Crohn’s disease severity. This observation is consistent with clinical studies showing that high levels of anti-*S. cerevisiae* antibodies are associated with a complicated clinical phenotype of Crohn’s disease and the need for surgery^28^,^29^.

In the present study, the analysis of *MBL2* polymorphisms revealed an association between three variants, rs930508, rs1800450 and rs5030737, and a reduction in mannose-binding lectin serum levels in Crohn’s disease patients. In addition, both homozygous and heterozygous *MBL2* mutations were associated with a decrease in mannose-binding lectin concentrations. Swale *et al*. showed that the presence of these variants (rs1800450 and rs5030737) was the major contributing factor for lower mannose-binding lectin concentrations^30^. However, we found that although the *MBL2* variant rs5030737 was associated with a low level of MBL-MASP functional activity, this variant was related to high anti-*S. cerevisiae* antibody levels in Crohn’s disease patients. These data corroborate previous observations, which showed that patients with low serum mannose-binding lectin or mannose-binding lectin deficiency were more often anti-*S. cerevisiae* antibody-positive than patients with normal levels of mannose-binding lectin^20^,^31^. The variant rs5030737 was found to be related to Crohn’s disease in a paediatric cohort^19^. In addition, Schoepfer *et al*. showed that a low mannose-binding lectin serum level was highly associated with complicated Crohn’s disease^31^. Altogether, our data emphasise the role of the rs5030737 variant in MBL-MASP functional activity and suggest that this variant affects the binding of mannose-associated serine proteases to the collagen-like domain of mannose-binding lectin. This may alter the innate immune response and increase the risk of developing a complicated Crohn’s disease phenotype.

The *NOD2* gene is involved in the innate immune response and is highly associated with Crohn’s disease^32^. Crohn’s disease-associated *NOD2* polymorphisms exhibit a reduced capacity to activate NF-κβ following muramyl dipeptide stimulation, suggesting that the loss of *NOD2* activation promotes Crohn’s disease^33^. *NOD2* polymorphisms, rs2066844 (R702W) and rs2066845 (G908R and rs2066847 (l1007fs), are the most common genetic variants associated with an increased risk of Crohn’s disease^34^. In addition, these variants alter the structure of either the leucine-rich repeat domain of the protein or the adjacent region^3^. In the present study, we observed that Crohn’s disease patients carrying the *NOD2* rs2066844 variant had low functional activity of the MBL-MASP complex, suggesting that both the *NOD2* rs2066844 variant and impairment of the functional activity of the MBL-MASP complex could affect the innate immune response against pathogens, leading to an increased risk of Crohn’s disease^35^,^36^.

In conclusion, although mannose-binding lectin serum levels did not vary significantly between Crohn’s disease patients and healthy controls, we selected 69 Crohn’s disease patients for a preliminary polymorphism analysis who reflected the characteristics of the original cohort in terms of the diversity of clinical phenotypes, age and sex. This made it possible to explore multiple genetic targets with a more focused approach (Supplementary data). The number of patients with the B3 phenotype was very low in our initial cohort (n = 40). We intend to increase our statistical power by including new Crohn’s disease patients with the B3 phenotype. Overall, our findings provide evidence that Crohn’s disease patients with severe clinical phenotypes have an impairment of MBL-MASP functional activity and that this defect is associated with *MBL2* and *NOD2* variants. This study will enable us to determine the relationship between MBL2 and NOD2 in Crohn’s disease and the way in which each affect the other by studying the signalling pathways.

## Methods

### Study population

The Crohn’s disease patients included in this study had previously been included in the MINOTOR cohort study at Lille University Hospital. At inclusion, the patients underwent a thorough clinical and laboratory examination. The diagnosis of Crohn’s disease was based on standard endoscopic, histological and radiographic findings^37^. The clinical phenotype of Crohn’s disease was determined according to the Montreal classification on the basis of age at onset (A), disease location (L) and behaviour (B)^38^. B1 corresponds to non-stenosing, non-penetrating disease, B2 to stenosing behaviour and B3 to penetrating behaviour. Sixty-nine Crohn’s disease patients (42 females/27 males; age at diagnosis: 10–50 years; B1 = 37, B2 = 16, B3 = 14, unclassified = 6) were included in the cohort. Thirty healthy control subjects (14 females/16 males; age 19–40 years) were also included. All healthy controls were free of symptoms and had a normal clinical examination. Details of the clinical phenotype of the Crohn’s disease patients including: age at diagnosis (A1: <16 years, A2: 17–40 years, A3: >40 years), disease location (L1: ileal, L2: colonic, L3: ileocolonic) and behaviour (B1: non-stricturing, non-penetrating, B2: structuring, B3, penetrating) are shown in Table 4.

All subjects were informed about the study and gave their written consent to participate. The study protocol was reviewed and approved by the Ethics Committees of Lille University Hospital (CP 05/86). The study was conducted according to the principles expressed in the Declaration of Helsinki.

### Measurement of mannose-binding lectin and anti-*S. cerevisiae* antibody concentrations

Mannose-binding lectin concentrations were measured by enzyme-linked immunosorbent assay according to the manufacturer’s instructions (BioPorto, Denmark) and are expressed as ng/mL. A concentration of <500 ng/mL was considered to represent mannose-binding lectin deficiency, low levels were 500–1000 ng/mL, normal levels were 1000–4000 ng/ml and a high mannose-binding lectin level was >4000 ng/mL.

Anti-*S. cerevisiae* antibodies were also detected by enzyme-linked immunosorbent assay (IBDX gASCA; Glycominds, Israel)^39^. Briefly, 50 μL of 1:100 diluted serum was added to the coated wells. Absorbance was read at 450 nm (reference filter, 620 nm) in a microplate reader (Bio-Rad) after addition of tetramethylbenzydine^40^. Results are expressed as arbitrary units (AU). Crohn’s disease patients were declared anti-*S. cerevisiae* antibody-positive when serum levels were >50 AU.

### Assessment of the functional activity of the MBL-MASP complex

Activity of the MBL-MASP complex in serum was determined using a modified version of a method described previously^27^. This assay is based on the thrombin-like activity of mannose-associated serine protease to cleave thrombin substrate. Briefly, 96-well plates (Nunc-Immuno, Maxisorp, Germany) were coated with 50 μl/well of *S. cerevisiae* mannan (1 mg/mL). After incubation for 24 h at 4 °C, the plates were washed twice with wash buffer (20 mM HEPES, 140 NaCl, 0.1% Tween, pH 7.4) and then incubated for 4 h at 4 °C with 200 μL of blocking buffer (20 mM HEPES, 140 mM NaCl, 5 mM EDTA, pH 7.4). After several washes, 50 μL of serum sample was mixed with 50 μL of dilution buffer (HEPES 40 mM, NaCl 2 M, CaCl_2_ 10 mM, pH 7.4) and then added to each well for 1 h at 4 °C. After several washes, fluorogenic thrombin substrate (VPR-AFC; Sigma, SCP0216) was added to each well and the plate was incubated at 37 °C for 1 h in a spectrofluorometer to measure the fluorescence released by cleavage of the thrombin substrate every minute (excitation 395 nm, emission 500 nm). The results are expressed as a percentage of emitted fluorescence (EF)/ng mannose-binding lectin. All samples were tested in duplicate. The increased fluorogenic signal was directly proportional to the mannose-associated serine protease activity (Supplemental data). After optimisation of various parameters, in particular the duration of measuring MBL-MASP activity, there was a plateau of activity at 1 h and no further variation in enzymatic activity could be detected (Supplemental data).

### Sequencing analysis

DNA extractions were performed as described previously^10^. DNA was extracted using a commercial kit according to the manufacturer’s instructions (Kit Nucleon BACC3; GE Healthcare). AmpliSeq libraries were prepared using an ion AmpliSeq library kit 2.0 and ion AmpliSeq custom panel (Life Technologies). AmpliSeq technologies were used to design a custom NGS library including 110 amplicons in two pools, covering all targets of interest (19.87 kb covered at 99.42%). The targets of interest were the *MBL2, NOD2* and *MASP1* genes. The design is available in the supplementary data. 10 ng of each DNA sample was used as a template to prepare the library. Quality control of all libraries was performed with an Agilent bioanalyser using high sensitivity chips. Template dilutions were calculated after library concentrations were normalized to ~100 pM using an ion library equalizer kit (Life Technologies). Library templates were amplified clonally using an ion one touch 2™, following the manufacturer’s protocol. Recovered template-positive ion sphere particles were subjected to enrichment according to the manufacturer’s instructions. Samples were subjected to the ion PGM 200 sequencing v2 protocol using ion 318 v2 chips (Life Technologies). Thirty-two barcoded samples were loaded per chip to ensure an average depth of 1500. For data analysis, alignment of the sequences to the human genome build 19 reference genome and base calling were performed using Torrent Suite software. Identification of variants was performed with an ion torrent variant caller and coverage analysis was generated using coverage analysis plugins (Life Technologies). Allelic frequencies and their association with anti-*S. cerevisiae* antibody and mannose-binding lectin levels were determined with Haploview software^41^.

### Statistical analysis

Statistical analysis was performed using Prism 4.0 from GraphPad and XLSTAT. Data were analysed using either the Kruskal-Wallis or Mann-Whitney U test to compare pairs of groups and the Chi^2^-test for comparison of two independent groups with categorical data. Differences were considered significant when the *P* value was as follows: *P* < 0.05; *P* < 0.01; *P* < 0.001. Fitting the Hardy-Weinberg equilibrium, allelic frequencies and their association with Crohn’s disease or anti-*S. cerevisiae* antibody levels were determined with Haploview software.

## Additional Information

**How to cite this article**: Choteau, L. *et al*. Polymorphisms in the Mannose Binding Lectin Gene are Associated with Defective Mannose Binding Lectin Functional Activity in Crohn’s Disease Patients. *Sci. Rep.* **6**, 29636; doi: 10.1038/srep29636 (2016).

## Supplementary Material

Supplementary Information

Supplementary Information

## Figures and Tables

**Table 1:** Evaluation of mannose-binding lectin phenotype and genotype in relation to the Crohn’s disease phenotype.

*MBL2* rs5030737	Wild-type (G/G)	Heterozygote (G/A)	Mann-Whitney *P*(α = 0.05)
**Number (%)**	60 (87%)	9 (13%)	
**Behaviour,** ***n*** **(%)**			
** B1**	35 (58.3%)	2 (22%)	
** B2**	12 (20%)	4 (44.4%)	
** B3**	11 (18.3%)	3 (33.3%)	
** IBDU**	2 (3.3%)	–	
**ASCA level (AU)**	56.55	110.4	<0.05
**MBL level (ng/mL)**	3211.9	366.4	<0.0001
**MBL-MASP activity (%EF/ng of MBL)**	0.35	0.13	<0.05

IBDU: inflammatory bowel disease not identified; AU: arbitrary unit; ASCA: anti-*Saccharomyces cerevisiae* antibodies; MBL: mannose-binding lectin; MASP: mannose-associated serine protease; EF: emitted fluorescence.

**Table 2:** Relationship between the rs2066844 *NOD2* polymorphism and clinical phenotype of Crohn’s disease.

*NOD2* rs2066844	Wild-type (C/C)	Heterozygote (C/T)	Mann-Whitney *P* (α = 0.05)
**Number (%)**	50 (72.5%)	19 (27.5%)	
**Behaviour**			
** B1**	23 (46%)	14 (73.3%)	
** B2**	13 (26%)	3 (15.8%)	
** B3**	12 (24%)	2 (10.5%)	
** IBDU**	2 (4%)	–	
**ASCA level (AU)**	62.8	66.7	ns
**MBL level (ng/mL)**	3046.5	2299.46	ns
**MBL-MASP activity (%EF/ng of MBL)**	0.38	0.16	p < 0.05

IBDU: inflammatory bowel disease not identified; AU: arbitrary unit; ASCA: anti-*Saccharomyces cerevisiae* antibodies; MBL: mannose-binding lectin; MASP: mannose-associated serine protease; EF: emitted fluorescence.

**Table 3:** Frequency of *MASP1* and *NOD2* polymorphisms in 69 Crohn’s disease patients and 30 healthy controls.

SNP	Crohn’s disease patients vs. healthy controls			European allele frequency		Crohn’s disease association *P*	MBL-MASP activity *P*
	Wild-type	Heterozygous	Homozygous	Ref	Muted		
***MASP1***							
rs850312	C/C: 56.5 *vs. 54*	C/T: 27.5 *vs. 38*	T/T: 16 *vs. 8*	C: 66	T: 34		
rs72549154	C/C: 94 *vs. 88*	C/A: 6 *vs. 12*		C: 97	A: 3		
rs3774268	A/A: 64 *vs. 78*	G/A: 32 *vs. 19*	G/G: 4 *vs. 4*	A: 15	G: 85		
rs34090319	G/G: 48 *vs. 50*	G/GG: 46 *vs. 42*	GG/GG: 6 vs. *8*				
rs78008995	G/G: 87 *vs. 88*	G/T: 13 *vs. 8*	T/T: 0 *vs. 4*				
rs72549251	T/T: 97 *vs. 92*	T/C: 3 *vs. 8*					
rs16861895	C/C: 69 *vs. 35*	C/G: 28 *vs. 61*	G/G: 3 *vs. 4*				
rs16861896	G/G: 69 *vs. 35*	G/A: 28 *vs. 61*	A/A: 3 *vs. 4*				
rs72549254	G/G: 69 *vs. 35*	G/A: 28 *vs. 61*	A/A: 3 *vs. 4*				
***NOD2***							
rs2066844 (R702W)	C/C: 72 *vs. 92*	C/T: 28 *vs. 8*		C: 95	T: 5	0.0518	<0.05
rs2066845 (G908R)	G/G: 88 *vs. 88*	G/C: 12 *vs. 12*		G: 99	C: 1		
rs2066847 (l1007fs)	C/C: 75 *vs. 96*	C/CC: 22 *vs. 4*	CC/CC: 3 *vs. 0*			0.0177	
rs2076753	G/G: 35 *vs. 50*	G/T: 36 *vs. 46*	T/T: 29 *vs. 4*			0.0119	
rs2067085	C/C: 55 *vs. 35*	C/G: 32 *vs. 54*	G/G: 13 *vs. 11*	C: 57	G: 43		
rs2066842	C/C: 33 *vs. 58*	C/T: 38 *vs. 38*	T/T: 29 *vs. 4*	C: 76	T: 24	0.002	
rs2066843	C/C: 33 *vs. 58*	C/T: 38 *vs. 38*	T/T: 29 *vs. 4*	C: 76	T: 24	0.002	
rs1861759	T/T: 56 *vs. 31*	T/G: 30 *vs. 54*	G/G: 13 *vs. 15*	T: 58	G: 42		
rs5743291	G/G: 91 *vs. 85*	G/A: 7 *vs. 15*	A/A: 1 *vs. 0*	G: 90	A: 10		
rs1077861	A/A: 55 *vs. 4*	T/A: 33 *vs. 69*	T/T: 12 *vs. 27*				

SNP: single nucleotide polymorphism; MBL: mannose-binding lectin; MASP: mannose-associated serine protease.

**Table 4:** Clinical characteristics of the Crohn’s disease patients.

	Crohn’s disease patients (*n* = 69)
**Mean age of onset (years)**	23
**Female/male**	42/27
**Montreal classification**	
16-years (A1)	17 (24.6%)
16–40-years (A2)	48 (69.6%)
>40-years (A3)	3 (4.3%)
Terminal ileum (L1)	15 (21.7%)
Colon (L2)	14 (20.3%)
Ileocolon (L3)	34 (49.3%)
Non-stricturing/non-penetrating (B1)	37 (53.6%)
Stricturing (B2)	16 (23.2%)
Penetrating (B3)	14 (20.3%)
**Perianal lesions**	23 (33.3%)
**Surgery**	42 (60.9%)
**Predisposed subjects**	14 (40.6%)

Values shown are n, or n (%).

A: age; L: disease location; B: behaviour.
