# Corrigendum: Polymorphisms in the Mannose-Binding Lectin Gene are Associated with Defective Mannose-Binding Lectin Functional Activity in Crohn’s Disease Patients

**DOI:** 10.1038/srep33426

**Published:** 2016-09-16

**Authors:** Laura Choteau, Francis Vasseur, Frederic Lepretre, Martin Figeac, Corine Gower-Rousseau, Laurent Dubuquoy, Daniel Poulain, Jean-Frederic Colombel, Boualem Sendid, Samir Jawhara

Scientific Reports 6: Article number: 29636; 10.1038/srep29636 published online: 07 12 2016; updated: 09 16 2016.

In the Introduction section of this Article, some instances of ‘inflammatory bowel disease’ are incorrectly written as ‘irritable bowel disease’.

