# Supplementary material for: Polymorphisms in the Mannose-Binding Lectin Gene are Associated with Defective Mannose-Binding Lectin Functional Activity in Crohn’s Disease Patients
Source: Sci Rep. 2016 Jul 12;6:29636. doi: 10.1038/srep29636 (PMC4940739; doi:10.1038/srep29636)
Supplement: Supplementary Information [file srep29636-s1.pdf]

Polymorphisms in the Mannose Binding Lectin Gene are Associated with Defective Mannose Binding Lectin Functional Activity in Crohn's Disease Patients

Laura Choteau; Francis Vasseur; Frederic Lepretre; Martin Figeac; Corine Gower-Rousseau; Laurent Dubuquoy; Daniel Poulain; Jean-Frederic Colombel; Boualem Sendid; Samir Jawhara

SUPPLEMENTARY DATA

Development of assay for MBL-MASP functional activity

To assess the functional activity of the MBL-MASP complex, cleavage of fluorogenic thrombin substrate was monitored over 1 h using a fluorometer (Fig. 1). Cleavage of this substrate reflects the MASP activity of substrates that bind to the collagen-like domain of MBL. We initially performed multiple pilot experiments using an MBL-positive serum from a healthy control subject (HC) with an MBL concentration >1000 ng/mL and another serum in which MBL was undetectable. After adding the fluorogenic thrombin substrate to both sera, we observed that cleavage of this substrate increased gradually in the MBL-positive serum when compared to the MBL-negative serum. In addition, the fluorogenic signal in the MBL-negative serum was comparable to that of PBS containing thrombin substrate only (Fig. 1A). To further assess the robustness and specificity of this assay in terms of the cleavage of thrombin substrate by the MBL-MASP complex from serum trapped on mannan-coated plates, we incubated the MBL-positive serum with either *S. cerevisiae* mannan (1 mg/mL), galactose (1 mg/mL) or sucrose (1 mg/mL) for 1 h at 4°C (Fig. 1B and 1C). In contrast to serum with either sucrose or galactose, serum with mannan did not produce any fluorogenic signal. This suggests that the addition of mannan, which binds to the MBL-MASP complex, prevents the complex from being trapped on the mannan-coated plates. These results confirm that the cleavage of thrombin substrate is specific to the MBL-MASP complex in serum trapped on mannan-coated plates (Fig. 1C).

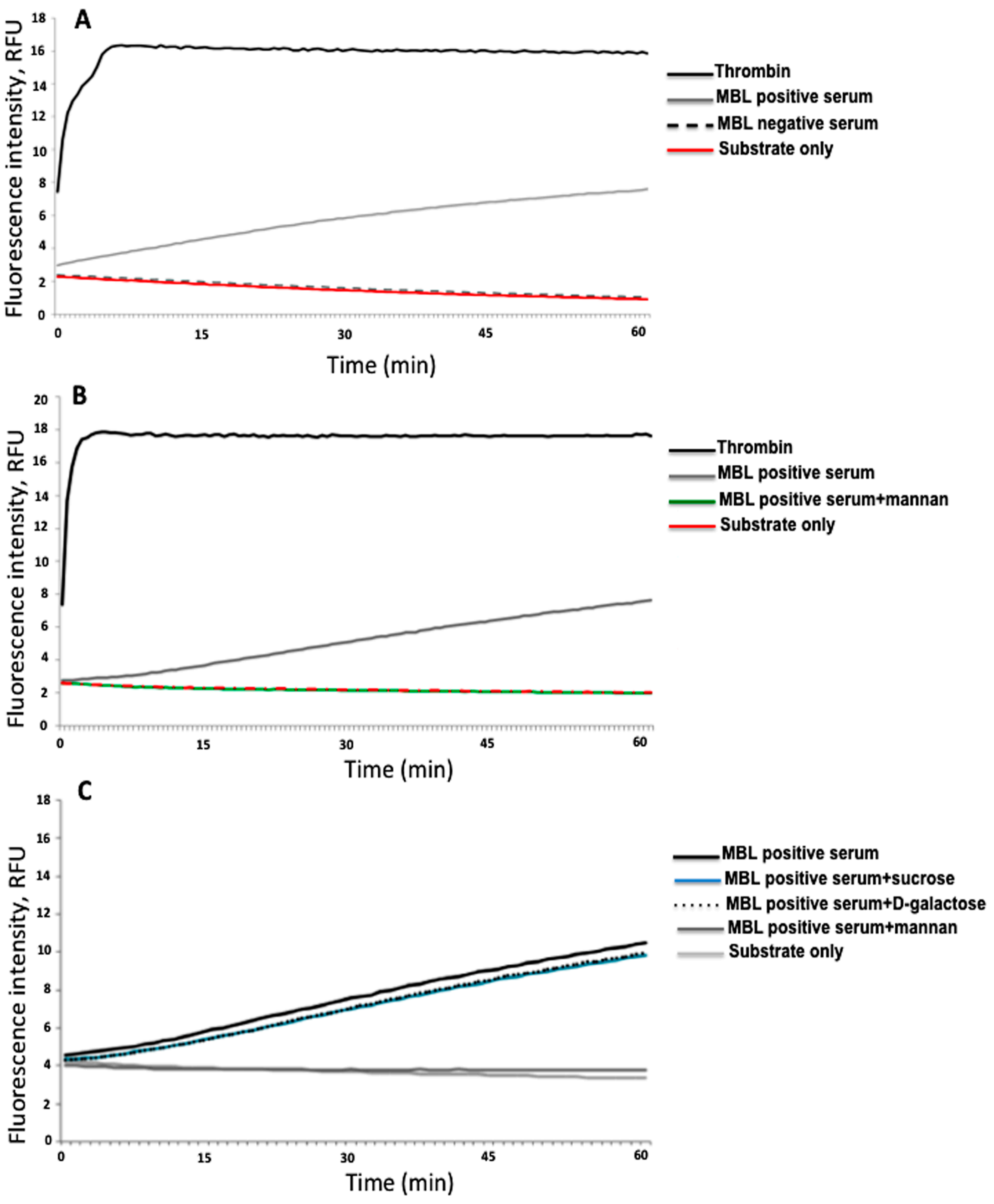

Figure 1: Cleavage of the fluorogenic thrombin substrate by the MBL-MASP complex. **(A)** Analysis of thrombin substrate activity in MBL-positive serum (gray line) and MBL-negative serum (black dashed line). Thrombin substrate without serum addition was used a positive control (black line). Substrate only was used as a negative control (red line). Cleavage of thrombin substrate was monitored for 1 h. **(B)** Analysis of thrombin substrate activity in MBL-positive serum containing mannan. **(C)** Analysis of thrombin substrate

activity in MBL-positive serum containing either mannan, sucrose or galactose. RFU, relative fluorescence units.

Alternative assay for MBL-MASP functional activity

In parallel to the fluorogenic thrombin assay, we performed another assay based on the release of ATP from platelets that had been exposed to the MBL-MASP complex trapped on mannan-coated plates. The concentration of ATP liberated from activated platelets was recorded by generated lights units (Fig. 2). This assay is based on the ability of MBL to bind to *S. cerevisiae* mannans through its carbohydrate recognition domain (CRD) and the ability of MASPs-associated with the collagen-like domain of MBL to activate platelets by releasing ATP. Multiple pilot experiments were performed using an MBL-positive serum from either HC or CD patients with an MBL concentration >1000 ng/mL and another serum in which MBL was undetectable. After addition of platelets to the MBL-MASP complex trapped on mannan-coated plates, we observed that ATP release increased gradually in the MBL-positive serum when compared to the MBL-negative serum. We then incubated the MBL-positive serum with either *S. cerevisiae* mannan (1 mg/mL) or with sucrose (1 mg/mL) for 1 h at 4°C. In contrast to serum with sucrose, serum treated with mannan showed a low level of ATP release, suggesting that the addition of mannan binding to the MBL-MASP complex prevented this complex being trapped on mannan-coated plates. This alternative method is consistent with the fluorogenic thrombin assay indicating that, like thrombin, the MBL-MASP complex also activates platelets (Fig. 2).

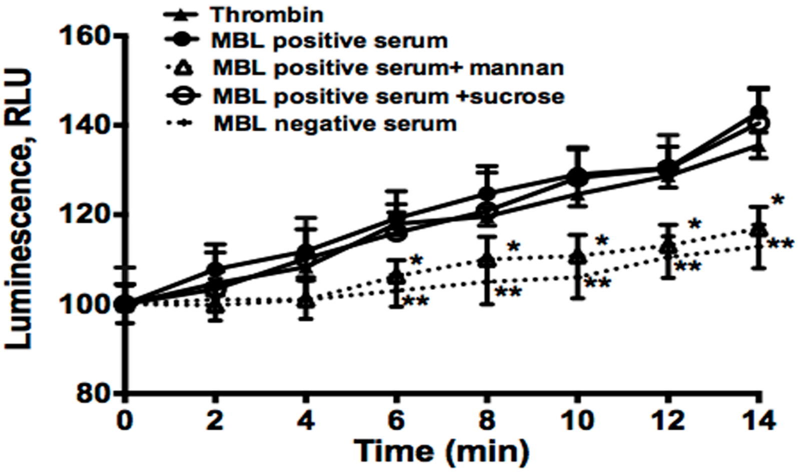

Figure 2: Platelet activation by the MBL-MASP complex. Changes in bioluminescence intensity corresponding to ATP release from activated platelets over the course of monitoring. Data are the mean ± SD from two independent experiments performed in quadruplicate. Thrombin (0.01 U/mL) was used a positive control. (***P*<0.05 for MBL-positive serum vs. MBL-negative serum, and **P*<0.05 for MBL-positive serum vs. MBL-positive serum + mannan). RLU, relative luminescence units.

Detection of C4b cleavage fragments

To confirm the functional activity results observed in the fluorogenic thrombin assay, we performed another assay based on cleavage of complement C4 protein to C4b fragments. This assay is based on the ability of MBL to bind to *S. cerevisiae* mannans through its CRD and the ability of MASPs-associated with the collagen-like domain of MBL to cleave the C4 protein to C4b fragments. We assessed the functional activity of the MBL-MASP complex in sera from 40 CD patients and 30 HC (Fig. 3). No significant difference was observed between HC and CD patients with B1, B2, or B3 phenotypes in terms of C4b level (Fig. 3A). We observed that C4b level was significantly correlated with the MBL concentration (Fig. 3B and 3C). These results are consistent with those obtained in the fluorogenic thrombin assay.

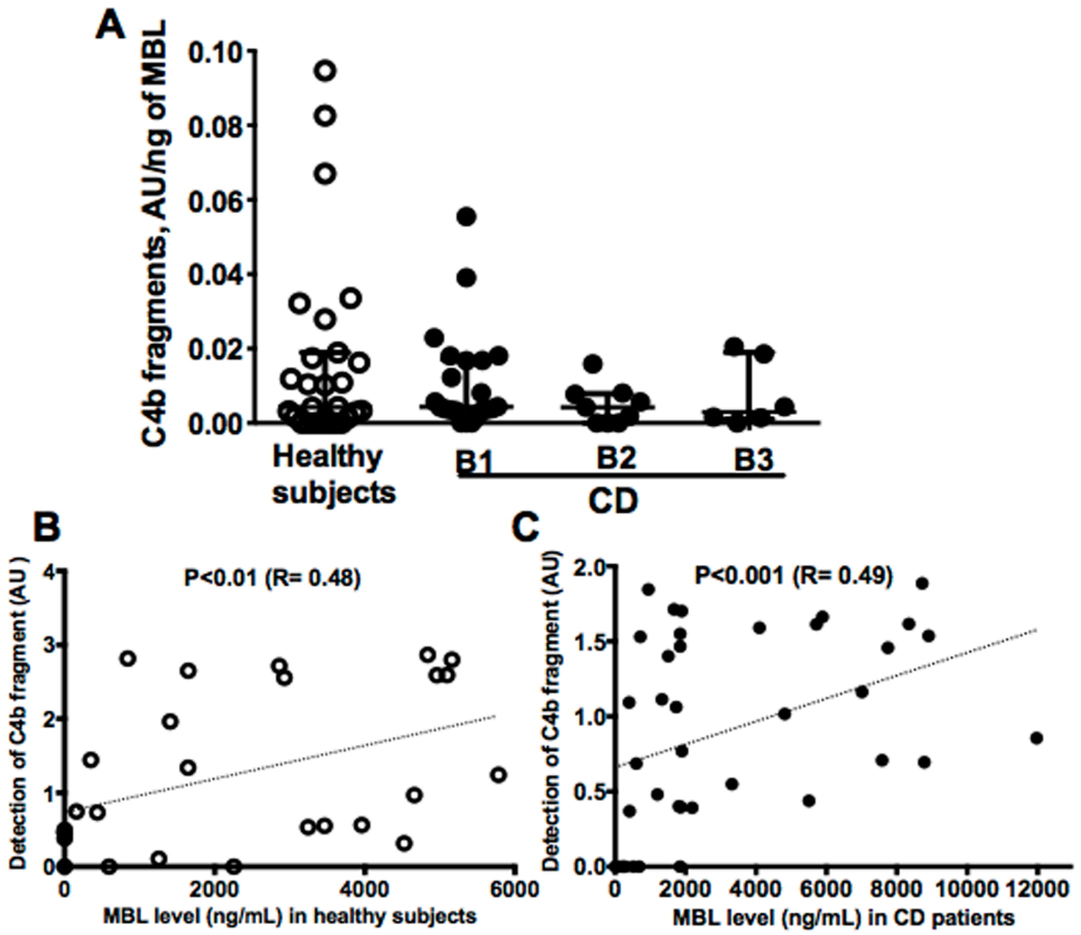

Figure 3: Determination of the functional activity of the MBL-MASP complex by cleavage of complement C4 to C4b fragments. **(A)** The functional activity of the MBL-MASP complex was determined in sera from 30 HC (open dot) and 40 CD patients (black dot). The activity assays were performed in duplicate for each sample. The results are expressed in arbitrary units (AU) per ng MBL. Data are the mean ± SD of two independent experiments. **(B and C)** Correlation between the cleavage of C4b fragments and MBL concentration in 30 HC ($P<0.01$, R=0.48), and 40 CD patients ($P<0.001$, R=0.49). AU, Arbitrary units.

Clinical phenotype of CD patients

A total of 225 patients with CD were initially enrolled in this study (mean age at diagnosis: 23 years). The clinical phenotype of these patients was classified according to the Montreal classification. We found that 49.8% of CD patients had the non-stricturing, non-penetrating

clinical phenotype (B1), while 32.4% had the B2 stricturing phenotype and 17.8% the B3 penetrating phenotype. The most common localization of CD in our patients was ileocolonic (56.4%). The 69 CD patients chosen for genotyping in this study reflect the same characteristics as the original cohort in terms of clinical phenotype, MBL levels and functional activity of the MBL-MASP complex. Thus, the percentages of patients with phenotypes B1, B2, or B3 were almost identical to those of the original cohort (53.6% B1, 23.2% B2, and 20.3% B3). Median MBL levels and functional activity were not significantly different between the initial cohort and the 69 CD patients (MBL levels: 2747.25 vs. 2840.8 ng/mL; functional activity: 0.31 vs. 0.26% FE/ng MBL) (Table 1).

Table 1: Clinical phenotype of CD patients

	**CD patients (*n*=225)**	**CD patients sequencing (*n*=69)**
Mean age at onset (years)	23	23
Sex, female/male	139/86	42/27
**Montreal classification**		
Localization, n (%)		
Ileal (L1)	51 (22.7%)	15 (21.7%)
Colonic (L2)	31 (13.8%)	14 (20.3%)
Ileocolonic (L3)	127 (56.4%)	34 (49.3%)
Behavior, n (%)		
Non-stricturing, non-penetrating (B1)	112 (49.8%)	37 (53.6%)
Stricturing (B2)	73 (32.4%)	16 (23.2%)
Penetrating (B3)	40 (17.8%)	14 (20.3%)

Perianal lesions	66 (29.3%)	23 (33.3%)
Surgery	129 (57.3%)	42 (60.9%)
Predisposed subjects	83 (36.9%)	14 (40.6%)
MBL concentration (ng/mL)	2747.25	2840.8
MBL-MASP activity (% EF/ng of MBL)	0.26	0.31

METHODS

Ethics Statement

All subjects gave their written informed consent to participate in the present study. The study protocol was reviewed and approved by the Ethics Committees of Lille University Hospital. In addition, the study was conducted according to the principles expressed in the Declaration of Helsinki.

Preparation of washed platelets

Whole blood was collected from healthy control subjects (HC). Platelets were isolated by differential centrifugation at increasing acceleration and then washed in modified Tyrode's buffer [1].

Bioluminescence detection of ATP release from activated platelets

To analyze ATP release, 96-well plates (Nunc-Immuno Maxisorp, Germany) were coated with 50 µl/well mannan from *S. cerevisiae* (1 mg/mL). After incubation for 24 h at 4°C, the plates were washed twice with wash buffer (20 mM HEPES, 140 NaCl, 0.1% Tween, pH 7.4) and incubated for 4 h at 4°C with 200 µL of blocking buffer (20 mM HEPES, 140 mM NaCl, 5 mM EDTA, pH 7.4). After several washes, 50 µL of serum sample was mixed with 50 µL dilution buffer (HEPES 40 mM, NaCl 2 M, $CaCl_2$ 10 mM; pH 7.4) and then added to each well for 1 h at 4°C. After several washes, washed human platelets (10^5 cells/well) in RPMI medium were added for 14 min. One hundred microliters of ATP assay buffer (ENLITEN ATP, Promega, France) was added to each well. ATP release from the platelets was monitored using a luminometer (Fluostar).

Cleavage of C4b fragments

This assay was performed as described previously [2,3]. Briefly, to assess the cleavage of C4 to C4b fragments, 96-well plates (Nunc-Immuno Maxisorp, Germany) were coated with 50 µl/well mannan from *S. cerevisiae* (1 mg/mL). After incubation for 24 h at 4°C, the plates

were washed three times with wash buffer (Tris-buffered saline with 0.05% Tween and 10 mM $CaCl_2$) and then incubated for 1 h at room temperature with 200 μL of blocking buffer (Tris-buffered saline with 1% bovine serum albumin (BSA)). After several washes, 50 μL of serum sample was mixed with 50 μL dilution buffer (Tris-buffered saline with 10 mM $CaCl_2$, 1 M NaCl, 0.05% Triton 100X, 0.1% BSA) and then added to each well for 90 min at 4°C. The dilution buffer contained 1 M NaCl to inhibit the C1q reaction and deposition of C4b, allowing only the MBL-MASP complex to bind to the mannan-coated plates. After several washes, TBS buffer containing C4 protein (diluted 1:3 with 10 mM $CaCl_2$) was added to each well. The plate was then incubated at 37° C for 90 min. Measurement of C4 cleavage to C4b from MBL-negative serum was used as a control. Detection of C4b was carried out using a specific biotinylated antibody (Bioporto, HYB162-02B) and streptavidin-peroxidase. Absorbance was read at 450 nm (reference filter, 620 nm) in a microplate reader (Bio-Rad) after addition of tetramethylbenzydine substrate. The results are expressed as arbitrary units (AU) or AU/ng MBL. All sera were assayed in duplicate.

REFERENCES

1 Byzova, T. V. & Plow, E. F. Networking in the hemostatic system. Integrin alphaiibbeta3 binds prothrombin and influences its activation. *The Journal of biological chemistry* **272**, 27183-27188 (1997).

2 Thiel, S., Moller-Kristensen, M., Jensen, L. & Jensenius, J. C. Assays for the functional activity of the mannan-binding lectin pathway of complement activation. *Immunobiology* **205**, 446-454, doi:10.1078/0171-2985-00145 (2002).

3 Petersen, S. V., Thiel, S., Jensen, L., Steffensen, R. & Jensenius, J. C. An assay for the mannan-binding lectin pathway of complement activation. *Journal of immunological methods* **257**, 107-116 (2001).
